# Recent advances in CRISPR-based functional genomics for the study of disease-associated genetic variants

**DOI:** 10.1038/s12276-024-01212-3

**Published:** 2024-04-01

**Authors:** Heon Seok Kim, Jiyeon Kweon, Yongsub Kim

**Affiliations:** 1 Department of Life Science, College of Natural Sciences, Hanyang University, Seoul, Republic of Korea; 2 Hanyang Institute of Bioscience and Biotechnology, Hanyang University, Seoul, Republic of Korea; 3 Hanyang Institute of Advanced BioConvergence, Hanyang University, Seongdong-gu, Seoul, Republic of Korea; 4 Department of Cell and Genetic Engineering, Asan Medical Institute of Convergence Science and Technology, Asan Medical Center, University of Ulsan College of Medicine, Seoul, Republic of Korea; 5 Stem Cell Immunomodulation Research Center, University of Ulsan College of Medicine, Seoul, Republic of Korea

**Keywords:** Gene therapy, Mutagenesis

## Abstract

Advances in sequencing technology have greatly increased our ability to gather genomic data, yet understanding the impact of genetic mutations, particularly variants of uncertain significance (VUSs), remains a challenge in precision medicine. The CRISPR‒Cas system has emerged as a pivotal tool for genome engineering, enabling the precise incorporation of specific genetic variations, including VUSs, into DNA to facilitate their functional characterization. Additionally, the integration of CRISPR‒Cas technology with sequencing tools allows the high-throughput evaluation of mutations, transforming uncertain genetic data into actionable insights. This allows researchers to comprehensively study the functional consequences of point mutations, paving the way for enhanced understanding and increasing application to precision medicine. This review summarizes the current genome editing tools utilizing CRISPR‒Cas systems and their combination with sequencing tools for functional genomics, with a focus on point mutations.

## Revolutionizing Precision Medicine: CRISPR-Cas Unlocks Genetic Mysteries

Genetic mutations, changes in our DNA, can cause many human diseases, but it’s hard to understand how they affect our cells. Traditionally, researchers have studied mutations that occur naturally, but this method has its limits. Genome editing methods, like the CRISPR-Cas system, allow for the study of disease-related genetic changes. In this review, Kim et al. outlined the integration of CRISPR-Cas tools with advanced sequencing technologies to explore how genetics influence disease. They comprehensively described both small-scale tests for individual changes and large-scale tests for the simultaneous analysis of many genetic changes. This review could help improve translational precision medicine (tailored treatments based on individual’s genetics), offering more personalized and effective treatments for various diseases. The study also shows the potential for future research in this field.

This summary was initially drafted using artificial intelligence, then revised and fact-checked by the author.

## Introduction

Many human diseases are attributed to genetic mutations, but comprehending how these mutations impact cellular phenotypes is limited by limited knowledge. Consequently, a significant number of mutations are classified as variants of unknown significance (VUSs), highlighting the crucial importance of understanding them for successful translational precision medicine^1,2^. Previously, researchers have relied on naturally occurring mutations found in existing biological samples, often through the use of genome-wide association studies (GWASs), to study their effects on phenotypes^3,4^. However, this approach was restricted to mutations present in specific samples. Genome editing methods, such as the CRISPR‒Cas system, facilitate the study of genetic variants associated with diseases. These methods are applicable to both protein-coding and noncoding regions of the genome, offering a comprehensive approach to understanding the genetic influences on disease^5^. Over time, various CRISPR‒Cas system orthologs have been identified, enabling successful genome editing in a wide range of organisms^6–14^. One of the key advantages of CRISPR technology lies in the ease of designing and synthesizing multiple guide RNAs (gRNAs), facilitating its application in high-throughput assays^15,16^. CRISPR-based high-throughput screens allow the simultaneous analysis of the functions of numerous genetic mutations. Furthermore, the concurrent development of diverse sequencing technologies, such as Illumina, Oxford Nanopore Technology (ONT)^17–21^, Pacific Bioscience (PacBio)^22^, and single-cell sequencing^23,24^, has opened up new avenues for the high-throughput evaluation of mutations. The integration of these technologies with CRISPR-based approaches allows researchers to comprehensively study the functional consequences of genetic mutations, paving the way for increased understanding and application of precision medicine.

Point mutations are the most common feature in human mutation databases, accounting for more than 50% of the mutations^1,2^. This review provides a summary of current genome editing tools utilizing the CRISPR‒Cas system and their combination with sequencing tools for functional genomics, with a focus on disease-associated point mutations.

### Tools for precise genome editing for functional genomics research

The CRISPR‒Cas system utilizes the Watson‒Crick base pairing code to recognize specific DNA sequences. Its target specificity is determined by two factors: the presence of a protospacer adjacent motif (PAM) sequence in the target DNA and the presence of a protospacer sequence in the gRNAs. PAM sequences are crucial for allowing Cas proteins to identify their target DNA, and assorted Cas variants have been engineered to recognize diverse PAM sequences, expanding the versatility of the system^25–29^. By simply altering the protospacer sequences in gRNAs, the target site of CRISPR‒Cas can be easily modified, making this system highly adaptable for functional genomics assessments. Furthermore, CRISPR–Cas9 techniques enable the generation of isogenic cell models that are genetically identical to wild-type cells except for the specific mutation of interest, providing valuable insights into the molecular mechanisms of genetic variations. Three primary classes of genome editing tools have been developed to date for precise functional genomics: nucleases, base editors, and prime editors.

#### Nucleases

**Fig. 1:**
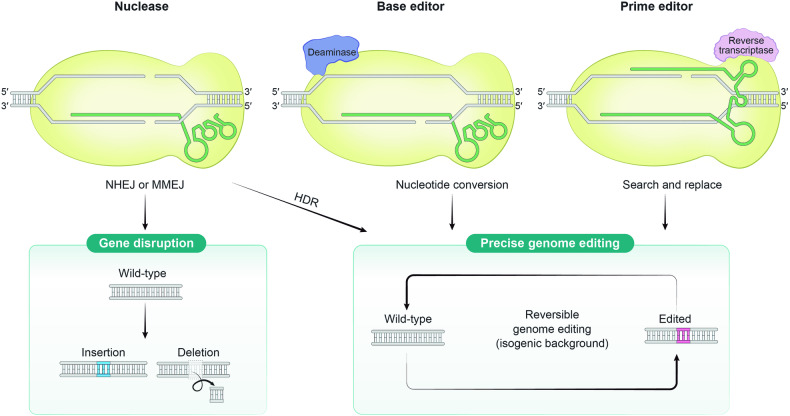
A schematic overview of genome engineering strategies for the functional study of disease-associated genetic variants. CRISPR‒Cas toolkits are classified into three categories on the basis of the use of nucleases, base editors, and prime editors. Red arrows indicate DNA breaks induced by CRISPR‒Cas toolkits. Nucleases can induce gene disruption by NHEJ-mediated mechanisms in gene knockout studies. Precise genome editing, which encompasses the introduction of disease-associated genetic variants and their correction, can be accomplished using nuclease-mediated HDR mechanisms, as well as through base editor-mediated and prime editor-mediated genome engineering.

Cas nucleases, exemplified by Cas9 and Cas12, are directed to specific DNA target sites in the genome through gRNAs and trigger DNA double-strand breaks (DSBs)^30^. Cellular DSB repair pathways, such as nonhomologous end joining (NHEJ) and homology-directed repair (HDR), then repair these breaks. In mammalian cells, nuclease-induced DSBs are predominantly repaired by the error-prone NHEJ pathway, leading to a mix of small insertion and deletion (indel) mutations at the target sites^31^. These indel mutations often cause frameshifts in coding sequences (CDSs), which disrupt genetic functions. Alternatively, DSBs can be accurately repaired by the error-free HDR pathway during specific cell cycle phases, namely, S and G2, allowing precise gene corrections with DNA donor templates^32,33^. While NHEJ-mediated gene disruption is highly efficient and is widely used to generate isogenic models that disrupt the gene of interest, facilitating comparisons between wild-type and knockout cells, HDR-mediated gene correction is commonly employed to understand the functional mechanisms of specific point mutations (Fig. 1). However, HDR has limitations for broader applications, including the need for additional DNA templates^30^, the potential for DSBs to induce undesired genomic alterations and activate the p53 response^34–36^, and variations in correction efficiency among different mammalian cell types^37^. Despite these challenges, the combination of Cas nucleases with multiple gRNAs enables multiplex gene knockout and the creation of large-scale structural variations in the genome, offering versatile tools for genomic manipulation and functional genomics research^38–40^.

#### Base editors

Base editors (BEs) are groundbreaking tools developed for precise nucleotide conversion, which is achieved by combining catalytically modified Cas proteins (such as Cas9-D10A nickases) with deaminases^41^. When a Cas protein and its gRNA recognize target DNA sequences, they form a single-stranded DNA (ssDNA) R-loop through gRNA hybridization with the target DNA strand. The deaminase domain then accesses the ssDNA R-loop and induces nucleotide conversion without causing DSBs. Two primary types of BEs have been developed: cytosine base editors (CBEs), which convert C:G to T:A base pairs, and adenine base editors (ABEs), which convert A:T to G:C base pairs^42–44^. Recently, engineered BEs, such as C:G to G:C base editors (CGBEs) and A:T to C:G base editors (ACBEs), have been further developed by fusing additional DNA repair factors to conventional BEs, significantly expanding their capabilities and application scope^45–48^. BEs can introduce and correct point mutations, which account for the majority of human somatic mutations associated with genetic diseases^49^ (Fig. 1). They can also be employed for targeted gene disruptions, e.g., the introduction of premature stop codons in coding sequences (CDS) or alterations in RNA splice-site motifs, all without causing DNA DSBs^50–53^. However, BEs have limited ability to induce specific types of nucleotide conversions and may also introduce undesired nucleotide changes within the base editing window^41^. Despite these challenges, BEs represent a promising tool for precise genome editing with significant potential for advancing our understanding and treatment of genetic diseases.

#### Prime editors

Prime editors (PEs) represent a significant advance in genome editing achieved by fusing a reverse transcriptase to catalytically impaired Cas9 proteins (Cas9-H840A nickases)^54^. These PEs are guided to target sites by a prime editing guide RNA (pegRNA), which contains template sequences for reverse transcription and protospacer sequences. The Cas9-H840A nickases cleave the target DNA strand, allowing the reverse transcriptase to synthesize a template DNA strand, thereby manipulating the target site^54^. This unique approach enables PEs to generate targeted genome modifications, including all types of substitutions and small indel mutations, to harness cellular DNA repair mechanisms (Fig. 1). Compared to Cas nucleases or base editors, PEs have a distinctive advantage: they can directly rewrite a target DNA without inducing DSBs or requiring donor DNA. Consequently, PEs offer high editing purity and target specificity. Recent studies have further increased prime editing efficiency through the engineering of PEs and the optimization of pegRNAs to increase expression, nuclear localization, and degradation resistance^55–60^. PEs are remarkably versatile methods for precise genome editing, facilitating functional genomics investigations of nearly all types of genetic variation with exceptional specificity. Notably, while Cas nucleases and base editors induce mutations mainly within protospacer regions, PEs can introduce modifications both in the 3’ regions of protospacers and within the protospacer sequences themselves. This unique feature enables PEs to correct multiple genetic variations, such as *KRAS* mutational hotspots, using a single pegRNA in a novel ‘one-to-many’ approach^61^. However, despite their distinct advantages, PEs currently exhibit lower efficiency than other genome editing technologies. Addressing this limitation will be crucial for the broader application of PEs in various fields and for unlocking their full potential in precision genome editing.

### Advantages of CRISPR-based genome editing for the functional study of genetic variants

**Fig. 2:**
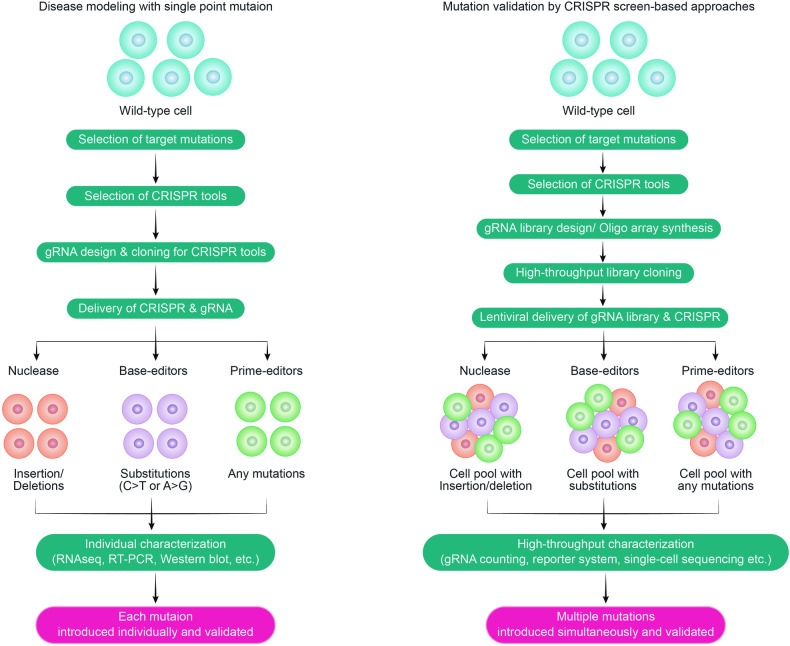
Comparison between single and multiplexed SNV characterization. This figure depicts a comparative analysis of two distinct approaches to SNV characterization. In the traditional method, individual clonal cells, each containing a specific SNV, are generated and separately characterized. In contrast, the high-throughput CRISPR screen-based multiplexed approach introduces multiple SNVs simultaneously and enables their collective analysis, greatly expanding the scale of SNV characterization.

CRISPR-based genome editing is unique in its ability to precisely target and modify specific locations within the genome, surpassing traditional methods that rely on the integration of external cDNA encoding genetic variants. This precision facilitates the generation of isogenic disease models, allowing nuanced and accurate analysis of phenotypic changes resulting from specific genetic mutations (Fig. 2). This targeted approach reduces artifacts from gene overexpression and advances the understanding of intricate gene regulatory mechanisms influenced by cellular signaling pathways^62^. Additionally, CRISPR technology is applicable beyond protein-coding sequences, enabling research on noncoding regions such as splicing junctions, untranslated regions (UTRs), promoters, enhancers, and other regulatory elements, as well as mutations in cellular noncoding RNAs and microRNAs^63–66^. This broadens the scope of genetic research and opens up new avenues for therapeutic interventions targeting genetic disorders at their root.

### Bulk CRISPR KO screens increase the throughput of functional genomics research

CRISPR-mediated multiplexed genome engineering has revolutionized gene function studies by enabling increased complexity and efficiency. In comparison to other genome engineering tools, such as zinc finger nucleases and TALENs, CRISPR offers distinct advantages for multiplexed engineering^30^. First, the design of gRNA, which determines the target gene, is remarkably straightforward. Second, gRNA molecules are compact in size, facilitating high-throughput synthesis. These features allow the implementation of high-throughput CRISPR screens. A widely utilized approach for conducting a CRISPR screen entails the delivery of a comprehensive genome-wide gRNA library into a large cell population via lentiviral vectors^67–70^. A distinct gRNA expression cassette is integrated into each cell to serve as both a barcode sequence and a specific knockout inducer. As a consequence, a pooled population of knockout cells is generated. These cells are subsequently exposed to diverse selective pressures, including cellular stressors, drugs, and toxins, which affect their overall fitness. After the selection process, the gRNA sequences within the cells are meticulously analyzed to identify genes that demonstrate growth advantages or disadvantages under the specific selection conditions. This detailed analysis enables the identification and characterization of genes that play a role in cellular responses and adaptations under the given selective pressures.

However, the scope of initial CRISPR screens is limited to phenotypes that exhibit a clear growth advantage or disadvantage. To overcome this limitation, researchers have incorporated various assays, including image-based assays, to expand the range of phenotypes that can be assessed^71,72^.

### Single-cell CRISPR KO screens enhance the granularity of functional genomics studies

Single-cell sequencing offers significant advantages and synergies when combined with CRISPR KO screens^73–75^. First, this approach allows individual analysis of each knockout cell within a pooled population. This capability provides valuable insights into the specific effects of each KO event. Second, this approach enables comprehensive analysis of the entire transcriptome of each knockout cell, shedding light on the global changes in gene expression resulting from the KO event. Previously, obtaining transcriptome information from KO cells required isolating individual KO cells and performing bulk RNA sequencing. With single-cell sequencing, it becomes possible to perform the same analysis on a pooled population of cells. This development enables researchers to obtain transcriptome information from a vast number of individual cells simultaneously, providing a comprehensive view of gene expression patterns across the pooled population. Consequently, by applying single-cell sequencing to pooled populations of knockout cells, researchers can achieve high-throughput analysis and gain deeper insights into the outcomes of each KO event, even in the absence of selective pressures or screening conditions. This combined approach significantly increases the resolution and understanding of the impact of CRISPR-mediated knockout on cellular processes.

Single-cell CRISPR screens involve the sequencing of both the transcriptome and gRNA from individual cells using droplet-based single-cell sequencing platforms. Unlike most coding genes that have a poly-A tail, gRNA lacks this feature. To address this, researchers have employed specific gRNA-encoding lentiviral vectors or incorporated specific reverse transcription primers to facilitate single-cell cDNA generation^20,75,76^. Subsequently, these single-cell cDNAs are sequenced using general short-read sequencing platforms. By comparing groups of cells with different gRNAs, the gene expression phenotype resulting from each KO event can be thoroughly analyzed. This integrated approach enables comprehensive elucidation of the functional consequences of gene knockouts at single-cell resolution. The adoption of long-read sequencing has further facilitated the analysis of differential transcript isoform usage resulting from gene knockout^20^.

### Bulk-level CRISPR screen for SNV characterization

Although Cas9 nuclease-based screens have significantly expanded the scalability of genetic studies, their applications have been focused on gene KO studies^20,67–77^. This limitation is noteworthy considering that more than half of human somatic mutations associated with genetic diseases are point mutations. Additionally, a considerable number of human single nucleotide variants (SNVs) have not been thoroughly studied and are classified as VUSs^1,2^. Therefore, it is crucial to identify the phenotypic effects of multiple human SNVs and address this gap. The development of strategies and technologies that enable the investigation of the functional consequences of SNVs will provide valuable insights into their effects on cellular processes and disease development (Fig. 2). By understanding the phenotypes associated with specific SNVs, researchers can better assess their clinical significance, inform diagnostics, and guide therapeutic interventions.

Prior to the advent of CRISPR base editors, Findlay et al. ^78^ developed a CRISPR-based saturation genome editing method for characterizing single nucleotide variants (SNVs) (Table 1). They employed Cas9 nuclease-mediated multiplex homology-directed repair (HDR) to generate all possible single-nucleotide variants (SNVs) at the target locus and then analyzed their functional impact. The Cas9-guide RNA complex introduces double-strand breaks (DSBs) at the target site, and these DSBs are repaired through HDR using a complex library of donor templates containing all possible SNVs. This process generates a pooled library of cells harboring diverse SNVs, which can be utilized for functional screening. This pioneering method was successfully applied to the accurate classification of nearly 4000 *BRCA1* variants^79^. Radford et al. ^80^ applied this approach to characterize 12,776 *DDX3X* variants and identified 3432 functionally abnormal variants, demonstrating its potential for large-scale variant characterization (Table 1).

**Table 1:** Comparison of multiplexed SNV characterization studies.

Investigated Genes	SNV introduction	Investigated SNV types	scRNAseq Method	Sequencing platform	SNV detection method	Analyzed features	Data analysis	Number of hits	Study
Genome-wide ( ~ 17,000 genes)	BE3	Premature termination codon	N/A	Illumina short-read	Indirect, sgRNA detection	Cellular fitness on 6-TG treatment	gRNA counting by custom script	1 gene	Kuscu et al. ^50^
*BRCA1* (~4000 SNVs)	Cas9 mediated HDR	Point mutations	N/A	Illumina short-read	Direct, mRNA sequencing	Cellular fitness	Custom python script	400 mis-sense SNVs	Findlay et al. ^79^
*DDX3X (12,776 nucleotide variants)*	Cas9 mediated HDR	Point mutations	N/A	Illumina short-read	Direct, gDNA sequencing	Cellular fitness	Custom R script	3432 functionally abnormal variants	Radford et al. ^80^
*BRCA1* (745 gRNAs)	BE3	Point mutations	N/A	Illumina short-read	Indirect, sgRNA detection	Cellular fitness on olaparib	MAGeCK	27 known mutations 4 VUSs	Kweon et al. ^88^
86 DNA damage response genes (~37,000 gRNAs)	BE3	Point mutations	N/A	Illumina short-read	Indirect, sgRNA detection	Cellular fitness on DNA damage	MAGeCK	~2000 gRNAs affecting DNA damage response	Cuella-Martin et al. ^89^
3584 ClinVar variant genes (~68,526 gRNAs)	BE3.9max	Point mutations	N/A	Illumina short-read	Indirect, sgRNA detection	Cellular fitness on various condition	gRNA counting using PoolQ tool and custom statistics	75 hit genes in the cisplatin screens	Hanna et al. ^90^
298 genes (~200,000 gRNAs)	Various CBEs	Point mutations	N/A	Illumina short-read	Indirect, surrogate target	Cellular fitness	Custom script	~5795 gRNAs affecting fitness	Sanchez-rivera et al. ^93^
29,060 cancer-related transition mutations	AncBE4max, ABEmax	Point mutations	N/A	Illumina short-read	Indirect, surrogate target	Cellular fitness	Custom script	18 outgrowing 157 likely outgrowing mutations	Kim et al. ^92^
*MAP2K1, KRAS, NRAS* (420 gRNAs)	BE3	Point mutations	CROP-seq	Illumina short-read	Indirect, sgRNA detection	Single-cell transcriptome	DROP-seq tools, CROP-seq software	Individual characterization of each mutations	Jun et al. ^95^
*TP53, KRAS* (100 variants per gene)	ORF library	Point mutations	Perturb-seq	Illumina short-read	Indirect, ORF barcode	Single-cell transcriptome	Cellranger, Seurat, Custom scripts	Individual characterization of each mutations	Ursu et al. ^94^
*TP53, SF3B1* (162 gRNAs)	AncBE4, ABEmax, CBE4max-SpG, ABEmax-SpG	Point mutations	Regular 5'	Illumina short-read, ONT long-read	Direct, full-length mRNA sequencing	Single-cell genotype Single-cell transcriptome	Cellranger, Seurat, Guppy, minimap, Custom scripts	Individual characterization of each mutations	Kim et al. ^21^

Base editor-based approaches enable more efficient and straightforward analysis of SNVs than HDR-based approaches^79,80^. Unlike HDR, which necessitates a repair template library in addition to a gRNA library, base editor-based methods streamline the analysis process. Hence, leveraging CRISPR base editors for SNV characterization will advance our comprehension of human genetic variation, offering insights into its implications for health and disease. A reliable gRNA design tool and efficient base editor constructs are indeed crucial for conducting high-throughput CRISPR base editor assays. In particular, the multiple-reporter-based gRNA efficiency prediction system has demonstrated its utility in increasing the success of CRISPR genome engineering^81–87^. Additionally, base editors with higher editing efficiency and broader PAM usage are valuable^28^.

CRISPR base editors have been adapted for the characterization of multiple SNVs, particularly nonsense mutations that introduce premature termination codons (PTCs). Researchers have conducted CRISPR screening studies utilizing cytosine base editors, which enable C-to-T substitutions and can introduce PTCs. Genome-wide analyses have demonstrated that cytosine base editors with the NGG PAM can potentially introduce PTCs into approximately 17,000 human genes^50,51^ (Table 1). While these studies have successfully introduced multiple expected PTCs at specific sites, it is important to note that the cellular consequences of these mutations are still similar to gene KO effects, similar to those in conventional CRISPR KO screens. The introduction of PTCs leads to the loss of functional protein products. While this approach provides valuable insights into the consequences of PTCs in specific genes, it is crucial to address a broader range of SNVs beyond nonsense mutations.

CRISPR base editor screens have been extended to target and investigate various missense mutations, which are commonly associated with cancer^88–91^. These studies have aimed to evaluate the functional consequences of multiple SNVs beyond nonsense mutations, particularly in cancer-related genes such as *BRCA1* and *BRCA2* (Table 1). By utilizing CRISPR cytosine base editors, researchers have been able to introduce specific nucleotide changes corresponding to known missense mutations found in cancer patients. This enable the examination of the resulting cellular phenotypes and assessment of the functional impact of these mutations on cancer-related pathways and processes. These expanded CRISPR base editor screens offer valuable insights into the functional consequences of missense mutations that have been categorized as VUSs and their potential associations with cancer development and progression. By characterizing the effects of specific SNVs through CRISPR base editing, researchers can elucidate the impact of these mutations on cellular processes and pathways involved in cancer. Moreover, experimentally subjecting cells with specific SNVs to anticancer chemical treatments can illuminate how the presence of certain mutations influences drug sensitivity. This knowledge is crucial in personalized medicine, as it helps identify patient-specific mutations that may affect the efficacy of targeted therapies or other interventions.

The interpretation of data obtained from CRISPR base editor screens poses challenges compared to that obtained from conventional CRISPR KO screens owing to the differences between CRISPR nucleases and base editors. First, in CRISPR KO screens, multiple gRNAs can be designed to knock out a gene, allowing a more robust interpretation of the cellular consequences of gene KO by individual gRNAs. However, when a CRISPR base editor is used to introduce specific SNVs, the design process is more constrained. To introduce a desired SNV, precise targeting of a specific site is necessary, which limits the ability to design multiple gRNAs that induce the same SNV. As a result, the interpretation of the results from CRISPR-based base editing screens often relies on a limited number of gRNAs, typically only one. Second, base editors can introduce multiple SNVs for each gRNA. When the target sequence contains multiple target bases, different substitution patterns can produce distinct amino acid changes. Consequently, analyzing the gRNA sequence alone does not fully capture the resulting SNVs introduced by base editors. Third, the efficiency of gene KO achieved by base editors is generally lower than the efficiency of introducing SNVs. Therefore the presence of a gRNA in a cell does not guarantee the successful introduction of the intended SNVs.

To overcome the challenges associated with interpreting CRISPR base editor screen data, two research groups recently introduced a reporter-assisted base editor screen method^92,93^. Instead of delivering only gRNAs to cells, they included both the gRNA and its corresponding target sites, which function as reporters of base editing events. By incorporating these target sites as reporters, the researchers were able to predict and quantify the base editing events that occurred at endogenous locations more precisely. This approach provides a more comprehensive assessment of editing efficiency and allows the precise identification and analysis of the introduced SNVs. For instance, Kim et al. identified 175 mutations in 160 genes as crucial drivers of cancer proliferation through a bulk screen using ABE and CBE^92^ (Table 1). These findings underscore the importance of functional studies in uncovering specific genetic mutations linked to diseases. Despite the advances enabled by the reporter-assisted base editor screen method, there are still certain limitations to consider. First, it is important to note that the base editing events observed in the reporter may not always be concordant with the endogenous homologous sites, as these events are independent of each other. Consequently, the reporter system might not accurately predict heterozygous mutations or fully reflect the complexity of the editing outcomes. Second, similar to other bulk CRISPR screening approaches, the scope of these methods is generally limited to phenotypes that can be assessed through simple growth advantages or disadvantages. This means that more intricate phenotypic effects resulting from SNVs, such as subtle changes in gene regulation or complex cellular responses, might not be captured by these screening methods alone.

### Single-cell-level approaches for SNV characterization

**Fig. 3:**
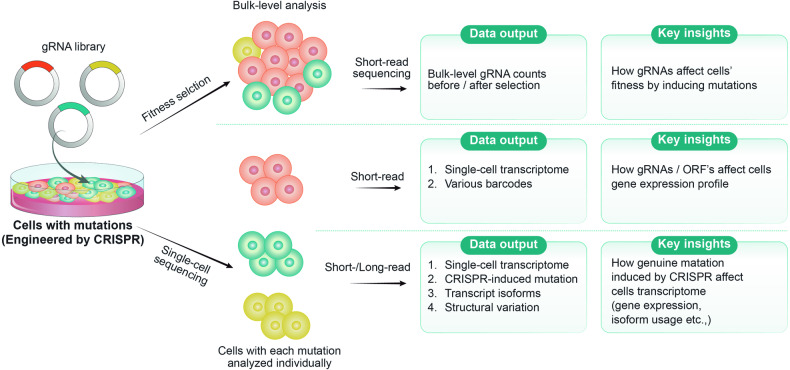
Strategies for multiplexed SNV characterization. This figure illustrates two main strategies for characterizing SNVs after high-throughput introduction. First, bulk analysis aided by gRNA tracking identifies SNVs with significant effects on cellular fitness. Second, single-cell analysis, including transcriptome analysis, provides deeper insights into the effects of individual SNVs, and the results can be extended by long-read sequencing to identify transcript isoforms and detect SNVs directly. These strategies enable comprehensive elucidation of SNV behavior, from overall fitness effects to precise gene expression patterns, thereby significantly advancing genetic research.

Single-cell RNA sequencing is a powerful technique that provides a more comprehensive understanding of the complex cellular responses induced by SNVs. By employing single-cell RNA sequencing to analyze pooled cells with multiple SNVs, researchers can gain insights into the impact of each SNV on the cellular transcriptome (Fig. 3). Ursu et al. reported a Perturb-seq-based technique that involves the overexpression of an open reading frame (ORF) library containing various SNVs (e.g., 100 for *TP53* and *KRAS*) in individual cells^94^ (Table 1). The authors evaluated the effects of these SNVs on the transcriptome using single-cell RNA sequencing. However, their study had several limitations. First, the overexpression of ORFs with SNVs differs from the introduction of endogenous mutations: these SNV-containing ORFs are not regulated by the endogenous promoter, and their expression levels can therefore differ from those of endogenous genes. This discrepancy might impact the interpretability of the results. Second, the endogenous wild-type genes continue to be expressed, potentially masking the effects of the SNVs. Finally, the barcode matching approach used in Perturb-seq, which employs DNA barcodes for each SNV, is known to be associated with a high frequency of barcode swapping, which can introduce errors during data analysis and lead to inaccurate interpretations. In a study conducted by Jun et al., a combination of base editor screening and single-cell RNA sequencing was employed^95^ (Table 1). The researchers utilized a cytosine base editor along with 420 gRNAs to introduce multiple endogenous SNVs and then performed single-cell RNA sequencing using the CROP-seq method. However, this study had limitations in terms of accurately determining the genuine SNVs and their effects. Although the researchers successfully introduced endogenous SNVs, they were unable to directly identify the specific SNVs that were introduced. Instead, they relied on detecting the gRNA present in each single cell, which allowed them to make educated guesses regarding which codon might have been edited. Consequently, they could not precisely interrogate the effects of each SNV. The inability to directly identify SNVs hindered the ability to establish a direct link between the introduced SNVs and the observed transcriptional changes. As a result, the conclusions drawn from this approach might lack the specificity and accuracy necessary to understand the true impact of SNVs on cellular responses. Moreover, both short-read-based assays lacked direct detection of SNVs at the single-cell level due to their limited read length, which was not able to cover both the cell barcode and SNVs simultaneously.

**Fig. 4:**
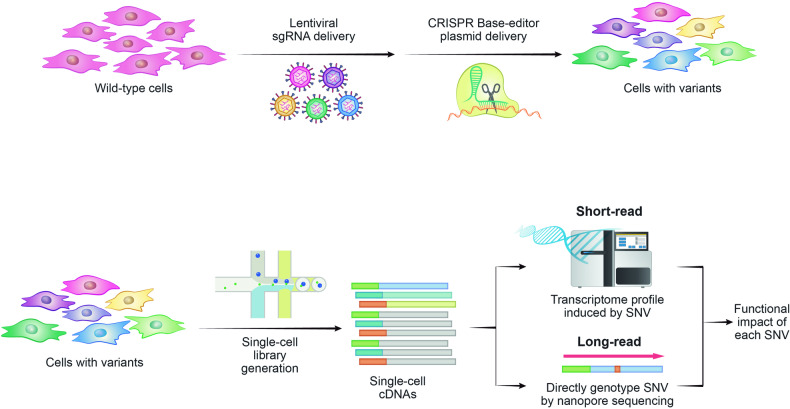
Schematic of TISCC-seq. This figure illustrates the detailed procedure of TISCC-seq. This approach seamlessly captures transcriptome profiles and genotypic information to enable the high-throughput evaluation of endogenous SNVs, surpassing the constraints of short-read methodologies.

The new technique called transcript-informed single-cell CRISPR sequencing (TISCC-seq) overcomes the limitations of short-read-based assays by the adaptation of long-read nanopore sequencing to single-cell base editor screens^21^ (Table 1). TISCC-seq utilizes various CRISPR base editor series and gRNA libraries to introduce multiple SNVs (Fig. 4). To comprehensively analyze the cellular landscape, TISCC-seq employs both short-read and long-read sequencing platforms. Short-read single-cell RNA sequencing provides transcriptome profiles for each cell, following the conventional approach. TISCC-seq further incorporates single-cell long-read sequencing, which enables the direct detection of each SNV introduced by the CRISPR base editor. This direct detection eliminates the need to rely on guesses based on gRNA or barcodes, increasing the accuracy and specificity of SNV identification. By simultaneously capturing the transcriptomic profile and genotypic information from the same single cell, TISCC-seq facilitates the high-throughput evaluation of genuine endogenous SNVs. In this study, the authors successfully obtained complete transcriptome data from cells harboring 169 mutations. They systematically categorized these mutations into 5 with phenotypes resembling the wild-type phenotype and 69 that exhibited statistically significant functional alterations. This novel approach significantly expands the ability to study the functional impact of SNVs on cellular responses and provides a valuable tool for comprehensive single-cell analysis.

## Conclusion

The exploration of human genetic mutations and their impact on cellular phenotypes, particularly the understanding of variants of unknown significance (VUSs), is crucial for advancing translational precision medicine. The integration of CRISPR-Cas genome editing tools with advanced sequencing technologies represents a substantial advance that has significantly broadened our understanding of the genetic influences of diseases on both protein-coding and noncoding regions. The versatility of the CRISPR‒Cas system, characterized by the ease of guide RNA (gRNA) design and its suitability for high-throughput assays, has revolutionized the functional study of genetic mutations. This system enables both low-throughput assays for the analysis of individual variants and high-throughput assays for the massive parallel analysis of genetic variants. These developments have not only increased our ability to analyze numerous genetic mutations simultaneously but also facilitated a deeper understanding of their functional consequences. As we continue to elucidate the roles of these genetic factors, come closer to realizing the full potential of translational precision medicine to offer more personalized and effective treatment strategies for various human diseases. The development of CRISPR‒Cas systems and their integration with state-of-the-art sequencing tools stand as a testament to the relentless pursuit of scientific innovation in understanding and combating genetic diseases.
